# Lectin from *Crataeva tapia* bark exerts antitumor, anti-inflammtory and analgesic activities

**DOI:** 10.1007/s13659-011-0014-8

**Published:** 2011-09-30

**Authors:** Regina M. S. Araújo, Antônio F. M. Vaz, Jaciana S. Aguiar, Luana C. B. B. Coelho, Patrícia M. G. Paiva, Ana M. M. Melo, Teresinha G. Silva, Maria T. S. Correia

**Affiliations:** 1 Departamento de Bioquímica, Centro de Ciências Biológicas, Universidade Federal de Pernambuco (UFPE), Avenida Prof. Moraes Rego s/n, 50.670-420 Recife, PE, Brazil; 2 Departamento de Antibióticos, Centro de Ciências Biológicas, Universidade Federal de Pernambuco (UFPE), Avenida Prof. Moraes Rego s/n, 50.670-420 Recife, PE, Brazil; 3 Departamento de Biofísica, Centro de Ciências Biológicas, Universidade Federal de Pernambuco (UFPE), Avenida Prof. Moraes Rego s/n, 50.670-420 Recife, PE, Brazil

**Keywords:** *Crataeva tapia*, lectin, antitumoral, anti-inflammatory, antinociceptive

## Abstract

*Crataeva tapia* bark lectin was evaluated for its antitumor activity against sarcoma 180 in Swiss albino mice. The anti-inflammatory and analgesic properties were investigated in models of inflammation and nociception. The anti-inflammatory assay was induced by carrageenan induced peritonitis and the analgesic activity was induced by acetic acid-induced writhing response. The lectin presents low toxicity with a LD_50_ of 2,500 mg/kg body weight and significant antitumor activity causing inhibition of tumor growth. The lectin also promoted significant reduction (35.4%) in the number of neutrophil migration induced by carrageenan. Concerning its analgesic property, the lectin inhibits abdominal contractions induced by acetic acid. The current results revealed a lectin with significant antitumoral, anti-inflammatory and antinociceptive activities. Further investigations to unveil the exact mechanisms are needed.

## Introduction

Plants have been a source of medicines for humans since pre-history and it is notable that in the last four decades, there has been a resurgence of interest in the study and use of medicinal plants. Natural products derived from plants have been characterized and identified as new chemical compounds of therapeutic importance. Lectins are proteins that can recognize and reversibly bind to carbohydrates or other substances derived from sugars[1]. Nowadays, it is well known that despite their small size, sugars play roles in storage or in relaying information within or between cells. Due to their carbohydrate-specific interaction, lectins have been versatile and useful molecular tools for the study of glycoconjugates on the cell surface. Therefore are excellent candidates to be explored in cancer research and as therapeutics agents[2–5].

Inflammation is part of the complex biological response of vascular tissues to harmful stimuli, such as pathogens, damaged cells, or cancer. Inflammation is usually associated with pain as a secondary process resulting from the release of analgesic mediators[6]. Non-steroidal anti-inflammatory agents – NSAIDS have been prepared and marketed[6], however, these drugs are known to provoke gastrointestinal irritation[7]. Thus, recognition of inflammatory processes incidence caused by cancer robustly correlates with an increasing attention on safety and effectiveness of novel pharmaceuticals. The aim of the present study was to investigate the antitumoral, anti-inflammatory and antinociceptive properties of bark lectin from *Crataeva tapia* on experimentally induced tumors, inflammation and pain.

*To whom correspondence should be addressed. E-mail: terezacorreia.ufpe@gmail.com

## Material and methods

**Purification of Crataeva Tapia Bark Lectin.** *Crataeva tapia* bark was obtained through a sequential purification protocol according to Nascimento et al.[8]. Bark powder (10g) was mixed with150 mM NaCl (100 ml). An extract was obtained by agitation of the mixture overnight at 4ºC. The extract was filtered through gauze and underwent centrifugation at 4,000 g for 15 min. The supernatant was termed crude extract (CE). The reversed micellar system was constituted using the anionic surfactant, sodium di(2-ethylhexyl)sulfosuccinate in isooctane. Extraction and back-extraction procedures were performed as follows: (1) to buffered lectin preparations (3 ml) containing

30 mM NaCl, an equal volume (3 ml) of micellar phase (AOT in isooctane) was added and both phases were stirred for 5 min for protein extraction. The mixture was then centrifuged for 5 min at 3000 g, for phase separation. (2) After extraction, the separated micellar phase (2 ml), containing solubilized protein, was added to an equivalent volume of buffered aqueous solution containing KCl and 5% (v/v) of butanol was added to the system. The mixture was stirred for 5 min, centrifuged for 5 min at 3000 g for phase separation and the lectin recovered to the new aqueous phase.

**Animals.** Male albino Swiss mice (*Mus musculus*) weighing approximately 33 g (±50 days old) were obtained from the Bioterium of the Centro de Pesquisa Ageu Magalhães / FIOCRUZ and maintained under constant conditions (temperature: 23 ± 2°C, humidity: 40–60%, 12 h light/12 h dark cycle). The mice were allowed to access standard rodent diet (Purina®) and water *ad libitum.* All experiments involving male albino Swiss mice were approved by the Ethics Committee for Animal Experimentation of the Biological Sciences Center of the Federal University of Pernambuco, Brazil (CEEA – UFPE-Ofício n° 40/06).

**Brine Shrimp Lethality Activity.** The assay was modified from Meyer et al.[9]. A*rtemia salina* eggs (25 mg) were hatched in natural seawater at temperatures of 20 to 30°C. The pH was adjusted between 8.0 and 9.0 using $Na_2CO_3$ to avoid risk of death to the larvae by decrease of pH during incubation[10]. *A. salina* bioassay used 5,000 ppm CrataBL (*Crataeva tapia* Bark Lectin) stock solution prepared in seawater: stock solution was diluted (110–50 ppm) in vials containing 5 ml of seawater and 15 *A. salina*. The control group was placed in diluted stock solution. Both groups were maintained in the solutions for 24 h. The survival rate was determined and used to estimate $LC_{50}$. The assays were carried out in triplicate. The collected data were computerized and the $LC_{50}$ value estimated using the statistical method of probity[11].

**Acute Oral Toxicity.** CrataBL was evaluated following the steps of Guideline 423[12], using male mice. The mice were prevented from eating overnight prior to the experiment. Three animals for each preparation were orally given a single dose starting at 300 mg/kg (mg of product/kg of body weight) and observed for 14 days (during the first hour after treatment and then once per day). CrataBL were dissolved in a 0.9% sterile NaCl solution and the calculation of the exact dose for each mice was based on individual weights. This first step was repeated in the same way for confirmation of results and a higher dose (2000 mg/kg) was administrated, both using different male mice.

**Antitumor Activity.** The *in vivo* antitumor activity of CrataBL against Sarcoma-180 was carried out in male mice (six animals per cage). CrataBL and MTX (methotrexate) dosages were calculated according to animal body mass index (20 mg/kg for CrataBL and 2.5 mg/kg for MTX). Malign tumor cells (Sarcoma-180) from tumor carrier animals were used after 8 days of the implantation. Donor mice were anaesthetized for tumor suctioning and the ascetic form of the tumor was introduced under the right axilla of the receptor animals. The treatment, by via the peritoneum, was begun 24 h after implantation and continued for 7 days. The negative control group received only the vehicle (saline solution) and the standard control (positive) received MTX as referential antitumor drug. On the eighth day, the animals were sacrificed by cervical dislocation and solid tumors excised and weighed. Tumor inhibition was expressed as the mean of tumor weight for the treated animal group (T) in comparison to the untreated control group (C). The tumor inhibition was then calculated according to: percentage tumor inhibition = ((C-T)/C)×100. Animal experiments were performed according to the NCI protocol[13].

**Anti-inflammatory Assay – Carrageenan-Induced Peritonitis.** Saline (0.15 M NaCl) solution (control), dexamethasone (2 mg/kg), piroxican (10 mg/kg) and indomethacin (10 mg/kg), as standard drugs, and CrataBL (30mg/kg), as the test drug, were administered via the peritoneum to the corresponding groups (6 animals per group). After 1 h, 0.25 ml carrageenan (1% in saline solution) injected intraperitoneally was used as a phlogistic agent. After 4 h the animals were sacrificed by cervical dislocation and immediately submitted to surgery for abdominal opening[14]. The peritoneal cavity was washed with 2 ml of saline solution with EDTA. Exudates were collected and the polymorphonuclear leukocyte (PMNL) count was determined in a Neubauer chamber after sample dilution in Turk solution (1:200).

**Analgesic Activity – Acetic Acid-Induced Writhing Response.** The response to an intraperitoneal injection of acetic acid solution, which manifests as a contraction of the abdominal muscles and stretching of the hind limbs, was evaluated using a method adapted from[15]. Animals (6 per group) were pretreated i.p. with CrataBL (30 mg/kg), vehicle (saline solution) or piroxican (10 mg/kg) and dipyrone (150 mg/kg) as standard drugs. After 1 h, a dose of 0.1 ml/10 g body weight of 1% acetic acid was injected i.p.. After 10 min, the number of writhings during the following 20-min period was counted. Inhibition percentage was calculated through the decrease of total writhing number in the treated groups against the control group.

**Statistical Analysis.** The results were expressed as mean ±S.E.M. (n=6) and statistically assessed using one-way ANOVA (Origin® 6.0). Significance was defined as $p$ values < 0.05.

## Results and Discussion

Compound CrataBL promoted *A. salina* mortality with a $LC_{50}$ of 71.73 $\mu$g/ml. According to Meyer et al.[9] crude extracts and pure substances with $LC_{50}$ value < 1000 $\mu$g/ml are toxic. The low value of $LC_{50}$ determined by *A. salina* bioassay indicates compounds with potential antineoplastic properties since there is good correlation between artemicide activity and cytotoxic activity[16]. At the doses of 300 mg/kg and 2,000 mg/kg, mice did not present weight loss or death. $LD_{50}$ cut-off of CrataBL was determined as 2,500 mg/kg body weight, resulting in both concentrations used being considered as safe (Category 5)[14].

Treatment of albino Swiss mice with CrataBL (20 mg/kg) significantly ($p < 0.05$) reduced the Sarcoma 180 volume as compared to the tumor volume of the control group (Figure 1). Antitumor activity of lectin has already been described. *Cratylia mollis* lectin promoted 41% tumor inhibition[3].

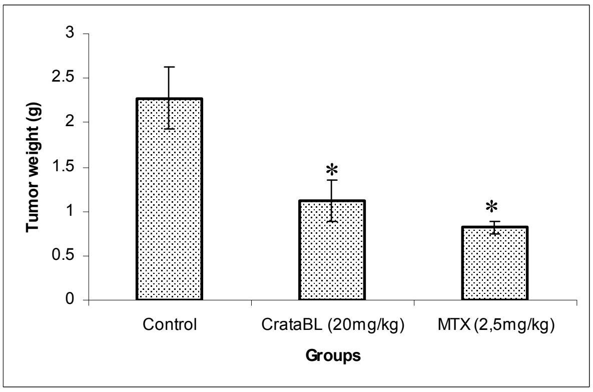

**Figure 1**. Effect of CrataBL and MTX [methotrexate] on growth of Sarcoma-180 in male albino Swiss mice. Each column represents the mean of six animals and vertical lines show the S.E.M. Asterisks denote the significance level in comparison to control value: *$P < 0.05$.

We have evaluated the putative anti-inflammatory activity of CrataBL using carrageenan-induced paw edema as an *in vivo* model of inflammation and evaluating the exudate from CrataBL treated and control groups. Our data showed that the lectin promoted significant reduction (35.4%) in the number of PMNL in the inflammatory exudate when compared with the control group (Figure 2). The paw edema model has been frequently used to assess the anti-edematous effect of natural products since carrageenan induces multi-mediated edema formation is characterized by intense neutrophil infiltrate and the release of inflammatory mediators such as polypeptide kinins, prostaglandins, and nitric oxide[17,18]. Anti-inflammatory lectins have already been revealed using the same experimental model used here. The lectin from marine alga *Hypnea cervicornis* significantly decreased neutrophil migration when injected into the peritoneal cavity of mice[5]. Santi-Gadelha et al. [4] demonstrated that the anti-inflammatory effect of *Araucaria angustifolia* seed lectin involved its carbohydrate binding sites. Piroxican and indomethacin are non-steroidal anti-inflammatory drugs commonly used to reduce fever, pain, stiffness, and swelling. It works by inhibiting the production of prostaglandins, molecules known to cause these symptoms. Dexamethasone is a potent synthetic member of the glucocorticoid class of steroid drugs. It acts as an anti-inflammatory and immunosuppressant. It is possible that CrataBL anti-inflammatory activity may be due to interference with the release of a cascade of inflammatory mediators since inhibition of leukocyte recruitment might prevent inappropriate inflammation[19].

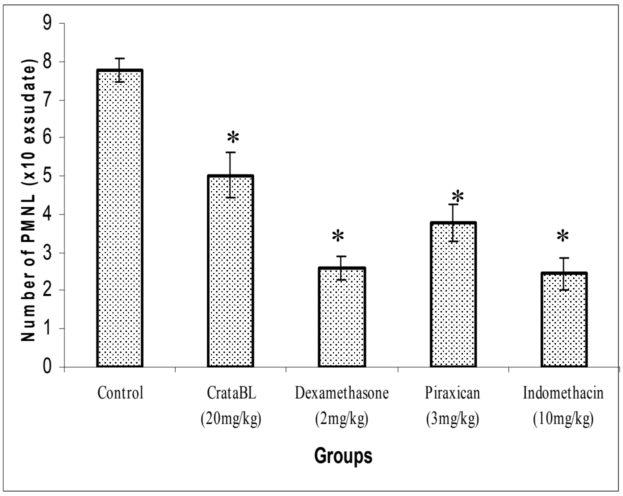

**Figure 2.** Effect of pre-treatment with dexamethasone, piroxican, indomethacin [standard drugs] and CrataBL on migration of polymorphonuclear leukocytes [PMNL] [number of PMNL/ml exudate] in carrageenan-induced peritonitis in mice. Each column represents the mean of six animals and vertical lines show the S.E.M. Asterisks denote the significance level in comparison to control value: *$P < 0.05$.

The results of acetic acid-induced writhing responses in mice treated with CrataBL and commercial antinociceptive drugs are showed in Table 1. It was found that CrataBL and drugs at the doses assayed caused a significant inhibition in the writhing responses when compared with the control. Researchers have aimed to elucidate different pharmacological effects of lectins related to their antinociceptive activity. Study with *Amansia multifida* lectin has demonstrated potent antinociceptive effects of both central and peripheral origin and it has been suggested that the analgesic effect possibly involved the participation of the opioid system[20]. Bitencourt et al.[5] demonstrated that the antinociceptive activity of lectin from marine alga *Hypnea cervicornis* may be related to inhibition of the release of mediators in response to acetic acid and that the activity involved the lectin carbohydrate-binding site.

**Table 1. Antinociceptive activity of CrataBL and analgesic drugs.**

| compound | dose | mean ± S.E.M. | protection (%) |
|---|---|---|---|
| control | | 64.4 ± 4.1 | |
| piroxican | 10 mg/kg | 44.7 ± 1.6* | 32.7 |
| dipyrone | 150 mg/kg | 19.8 ± 1.9* | 73.6 |
| CrataBL | 30 mg/kg | 42.0 ± 4.1* | 34.8 |

It is clear that there is a strong association between the inflammatory process and the development of pain[6]. Experimental studies have demonstrated that the inhibition of neutrophil migration reduces hyper-nociception induced by different inflammatory stimuli[21]. *Lonchocarpus sericeus* lectin decreased leukocyte migration and mechanical hyper-nociception by inhibiting cytokine and chemokine production [22]. CrataBL antinociceptive activity may be associated to its anti-inflammatory activity demonstrated here.

In summary, our work describes interesting biological properties of lectin from *Crataeva tapia* bark that not only displays a significant antitumor effect but also shows anti-inflammatory and antinociceptive activities. Further investigations to unveil the exact mechanisms are needed.

## Acknowledgments

This work was supported by the Conselho Nacional de Desenvolvimento Científico e Tecnológico (CNPq). The authors also wish to thank Ronaldo Celerino da Silva and Sílvia Rafaelli Ramos for their valuable technical assistance.

**Open Access** This article is distributed under the terms of the Creative Commons Attribution License which permits any use, distribution, and reproduction in any medium, provided the original author(s) and source are credited.
